# Understanding the release mechanisms and secretion patterns for glucagon-like peptide-1 using the isolated perfused intestine as a model

**DOI:** 10.1042/BST20241062

**Published:** 2025-01-31

**Authors:** Katrine D. Galsgaard, Ida M. Modvig, Jens J. Holst

**Affiliations:** 1 Department of Biomedical Sciences, Faculty of Health and Medical Sciences, University of Copenhagen, Copenhagen, Denmark; 2 Novo Nordisk Foundation Center for Basic Metabolic Research, Faculty of Health and Medical Sciences, University of Copenhagen, Copenhagen, Denmark

**Keywords:** enteroendocrine cells, glucagon-like peptide-1, gut hormones, intestine, model, perfusion

## Abstract

In the gastrointestinal (GI) tract, food is digested and absorbed while GI hormones are secreted from the enteroendocrine cells (EECs). These hormones regulate food intake, glucose homeostasis, digestion, GI motility, and metabolism. Although EECs may express more than a single hormone, the EECs usually secrete only one or a few hormones. The pattern of EEC secretion varies along the length of the GI tract as the different EEC types are scattered in different densities along the GI tract. Following bariatric surgery, a postprandial hypersecretion of certain GI hormones occurs which contributes to the postsurgery weight loss. Mimicking this postprandial hypersecretion of GI hormones by targeting endogenous EEC secretion, using specific modulators of receptors, ion channels, and transporters found on specific EECs, to induce weight loss is a current research aim. To achieve this, a more complete understanding of the release mechanisms, expression of receptors, transporters, and the secretion pattern of the different EEC types is needed. Using the vascularly perfused intestinal model, it is possible to obtain a detailed knowledge of these release mechanisms by evaluating the effects on secretion of blocking or stimulating specific receptors, ion channels, and transporters as well as evaluating nutrient handling and absorption in each of the different sections of the intestine. This mini-review will focus on how the isolated perfused intestine has been used in our group as a model to investigate the nutrient-induced release mechanisms of EECs with a focus on glucagon-like peptide-1 secreting cells.

## The enteroendocrine cells and their hormones

**Figure 1:**
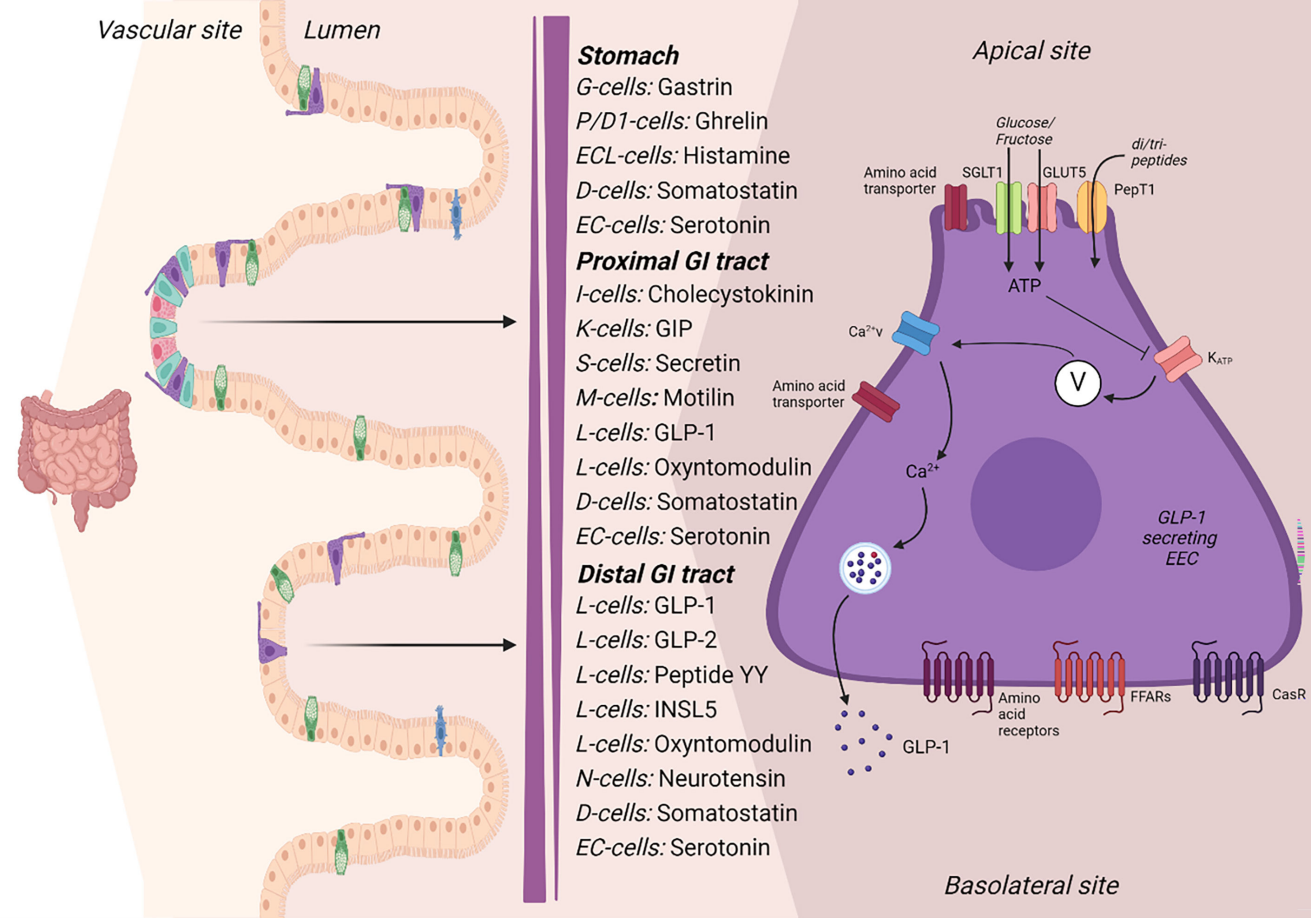
Hormone secretion in the gastrointestinal tract. Along the gastrointestinal (GI) tract, enteroendocrine cells (EECs) are scattered. In response to stimuli, the EECs secrete a range of GI hormones; here, the EEC cells and their secreted hormone are annotated according to the one cell–one hormone concept. However, most L-cells also secrete oxyntomodulin and Peptide YY. The glucagon-like peptide-1 (GLP-1) secreting EECs expresses a range of nutrient transporters and receptors as well as ion channels which in different ways respond to stimuli and result in GLP-1 release. GIP, glucose-dependent insulinotropic polypeptide; GLP-2, glucagon-like peptide-2; INSL5, insulin-like peptide 5; SGLT1, sodium glucose transporter 1; GLUT5, glucose transporter 5; PepT1, peptide transporter 1; FFARs, free fatty acid receptors; CaSR, calcium-sensing receptor. Illustration of mechanisms of GLP-1 secretion EEC released inspired by [1]. Created in BioRender. Galsgaard, K. (2024) https://BioRender.com/g68z042.

During digestion and absorption, a variety of hormones are secreted from the intestine in a defined postprandial pattern. These hormones include glucagon-like peptide-1 (GLP-1), glucagon-like peptide-2 (GLP-2), oxyntomodulin, glicentin, glucose-dependent insulinotropic polypeptide (GIP), cholecystokinin (CCK), secretin, neurotensin, and peptide YY (PYY) (Figure 1). Together these hormones function to regulate appetite, gastric emptying, GI motility, blood flow, and metabolism. The orexigenic hormones, ghrelin [2] and insulin-like peptide 5 [3], are secreted along with motilin [4] and possibly somatostatin [5] from the GI tract during the fasting state. The GI hormones that promote satiety and lower blood glucose levels have gained special attention due to their pharmacological potential in the treatment of obesity and type 2 diabetes. Importantly, GLP-1 reduces appetite and blood glucose levels, and GLP-1-based drugs are now used to treat obesity [6] and type 2 diabetes [7]. Indeed, several of the postprandially released GI hormones and their receptors are considered potential therapeutic targets, either alone or in combination, in the treatment of metabolic diseases [8].

The GI hormones are secreted from the enteroendocrine cells (EECs) which are scattered along the GI tract and constitute less than 1% of the epithelial cells of the GI tract [9,10]. Each EEC was previously thought to secrete only one specific hormone (the *one cell–one hormone* concept); however, recent research has shown that each EEC is capable of expressing more than just one hormone [10–17]; indeed, the co-expressed hormones have been found both in the same and in separate secretory granules within the EECs [18–20]. As mentioned, the pattern of EEC secretion varies along the length of the GI tract, resulting in some hormones (e.g., GLP-1) being potentially secreted along the entire GI tract and others mostly from the proximal (e.g., GIP and CKK) or distal (e.g., PYY) part of the GI tract [14]. Supporting the hormonal overlap within the EECs, fluorescence double staining demonstrated that most PYY-positive cells were also GLP-1 positive particularly in the distal part of the intestine, whereas in the proximal part, cells staining positive for only GLP-1 were common, pointing to the existence of different populations of GLP-1 expressing cells [21]. The co-expression of hormones seems to depend not only on the position of the EEC in the GI tract but also on the position of the EEC in the villus–crypt axis [21], which may relate to maturation of the EEC [12]. The characteristics of the varying EEC types may be influenced by the paracrine environment; and importantly, cells located in the proximal part of the intestine are responsible for the majority of nutrient absorption and are thus exposed to macronutrients (carbohydrates, proteins, and fat), whereas the colonic EECs are exposed to microbial metabolites and secondary bile acids [22], which may stimulate PYY and GLP-1 secretion, as indicated from studies of the perfused rat and mouse intestine [23,24]. Interestingly, although GLP-1 expressing cells are found in large numbers in the colon [25], it is not known to what extent and under which conditions colonic secretion contributes to the circulating hormone levels. In a study by Panaro et al., the distal gut was found to be important for the GLP-1 secretion in mice upon stimulation with a GPR119 agonist and melanocortin 4 receptor agonist (but not upon olive oil or arginine stimulation) [26]. However, the L-cell deletion included the ileal L-cells and therefore does not inform about the colonic L-cells. The EECs have been found to express a range of receptors and transporters suggesting an ability to respond to multiple stimuli [14,16]. Some of the receptors (such as certain bile acid receptors [27]) of the EECs are expressed on the basolateral side of the cells; this means that they may be targets for ligands/nutrients that are absorbed by the neighboring enterocytes [28,29]. Our knowledge and understanding of the EECs are far from complete. A detailed understanding of the different EEC types and their release machinery, expression pattern of receptors and transporters as well as a new classification system, replacing *the one cell–one hormone* concept, will be helpful in attempts to manipulate the secretion of specific GI hormones.

### Targeting enteroendocrine cell secretion

The most effective treatment of obesity is currently bariatric surgery in the form of Roux-en-Y Gastric Bypass (RYGB) or sleeve gastrectomy which results in a weight loss of appetite 20% to 30% [30], with RYGB generally resulting in a greater weight loss than sleeve gastrectomy [31]. Continued treatment (88 weeks) with the GLP-1- and GIP-receptor co-agonist, tirzepatide, may result in a weight loss of up to 25% [32], thus approaching that of bariatric surgery. It is currently being discussed whether the less invasive pharmacological treatment with GLP-1-based drugs is cost-effective compared with surgery in the long term [33]. Following RYGB and sleeve gastrectomy, a postprandial hypersecretion of the GI hormones, GLP-1, oxyntomodulin, PYY, and neurotensin [34] is generally observed as early as 2 days after surgery [35], while secretion of CCK, GIP, and secretin is moderately affected [36–40] and ghrelin [41] and perhaps gastrin [42] secretion is reduced. Sleeve gastrectomy and RYGB result in different postprandial GI hormone profiles postsurgery [40], with a more pronounced GLP-1 secretion following RYGB [40], thus somewhat different hormonal changes may be contributing to the appetite-lowering effect following the two procedures [40]. Following RYGB, accelerated nutrient absorption occurs [37,43], and food is delivered to a more distal part of the intestine where the majority of hypersecreting EECs are located, as reviewed in [44]. This may explain the increased hormone secretion. Postsurgery remodeling of the GI tract also occurs after RYGB, including villus hypertrophy possibly due to increased GLP-2 secretion [45,46], leading to more GLP-1 and PYY secreting cells. The hypersecretion of GI hormones, which is responsible for a large part of the weight loss, seems to be maintained long-term (up to 20 years) following RYGB [47,48]. In sleeve gastrectomy, the stomach is reduced which might also contribute to the observed weight loss via the simultaneous loss of ghrelin secretion [49].

For several years, it has been a goal to induce weight loss by mimicking the postprandial hypersecretion of GI hormones observed after bariatric surgery by targeting endogenous EEC secretion, as reviewed for GLP-1 secretion in [50]. To stimulate EEC hypersecretion, pre-meals or preloads of specific nutrients, and nutraceuticals, have been considered. However, the calorie content of these makes them a less desirable option in the treatment of obesity. Instead, it might be feasible to target EEC secretion using specific modulators of receptors, ion channels, and transporters potentially delivered to a specific part of the GI tract. As mentioned, GLP-1-based drugs are used to treat obesity [6] and type 2 diabetes [7], but transient GI events such as nausea and vomiting are reported [51]; however, the effect of these drugs on metabolic health (by reducing body weight and blood glucose levels and having cardio-, reno-, and neuro-protective effects [52,53]) generally outweighs the side effects. The side effects may be related to the observation that exogenous GLP-1 and GLP-1-based drugs appear to reach the circumventricular organs of the brain directly and from here activate neurons in various nuclei to reduce appetite [54–57]. By targeting endogenous GLP-1 secretion, these side effects could potentially be avoided as endogenous GLP-1 would mainly activate local sensory afferent nerves that via the nucleus of the solitary tract relay the signals to the appetite-regulating brain areas [58]. Targeting endogenous EEC secretion would also result in a range of GI hormones regulating appetite and glucose homeostasis including CCK, GIP, GLP-1, PYY, oxyntomodulin, and neurotensin being co-secreted in a manner depending on the targeting mechanism used. Another benefit of endogenous secretion of multiple GI hormones is that intestinal blood flow would be increased (a consequence of GIP [59] and GLP-2 [60] secretion) and epithelial barrier function would be improved (as a result of GLP-2 secretion [61]), which would potentially be beneficial in the treatment of obesity [62]. Malabsorption of certain vitamins and minerals resulting in nutritional deficiencies, observed after certain types of bariatric surgery as reviewed in [63] would also be avoided. A challenge when targeting endogenous GLP-1 secretion is that native GLP-1 is subject to immediate enzymatic degradation by dipeptidyl peptidase 4 (DPP-4) [64] and neprilysin [65] and negative feedback regulation mediated by somatostatin [66]. In principle, this could be overcome by co-treating with DPP-4 [67] and neprilysin inhibitors [68] or a somatostatin receptor antagonist [69], potentially making it possible to achieve sufficiently high endogenous GLP-1 levels to get the same effects as seen with GLP-1-based drugs. However, as endogenous GLP-1 acts locally immediately after secretion, endogenous hypersecretion may be beneficial even without the prevention of its degradation. To achieve endogenous hypersecretion of specific GI hormones, a complete understanding of the sensing mechanisms and release pattern of the different EEC types are needed; to achieve this, one cannot rely exclusively on cell lines, the integrated system which is preserved in the isolated vascularly perfused intestine must also be studied.

### Quantification of GI hormones using the isolated perfused intestine as a model

**Figure 2:**
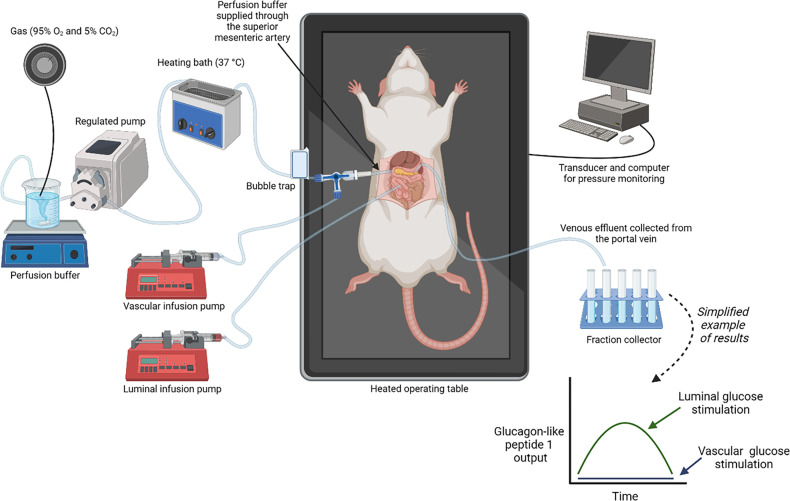
The isolated perfused rat intestine. This figure illustrates the setup for a perfusion experiment. The figure represents a further development of the figure shown in [70]. The simplified example of results is based on [71]. Created in BioRender. Galsgaard, K. (2024) https://BioRender.com/t19c527.

The isolated perfused intestine as a model, illustrated in (Figure 2), is described in detail previously [70]. In this model, rats or mice (thus enabling studies on genetically modified mice) are sedated, and a segment of the intestine (either the proximal or distal small intestine or the colon as exemplified in [72]) is isolated by restricting the blood supply to the remaining intestine. A catheter in the superior mesenteric artery is responsible for the vascular perfusion. Because the intestine is perfused through the superior mesenteric artery, which supplies only the distal duodenum and the remaining small intestine, the most proximal part of the duodenum is not perfused. The duodenum may be included but this requires perfusion via the aorta and ligation of the gastric vessels; however, in that case, an admixture of perfusion medium from the pancreas is difficult to avoid. A second catheter is placed in the portal vein and from here the venous effluent is collected frequently, for example, every minute or in shorter or longer time intervals yielding a conveniently large sample volume dependent on the flow rate. A third tube is placed in the intestinal lumen allowing luminal perfusion. The secretion of GI hormones can be quantified from the venous effluent using methods such as radioimmunoassay, enzyme-linked immunosorbent assay, and mass spectrometry, depending on the research question. As soon as a flow through the intestine is established, the animal may be euthanized. The intestinal cells (EECs, enterocytes, paneth cells, goblet cells, tutf cells, M cells, and Cup cells) are kept alive in their natural environment while cell polarity, peristaltic movements, and local nerve and blood vessel supply are kept intact. Compounds that activate or inhibit specific transporters, ion channels, and receptors can now be administered directly either luminally or vascularly, at the desired concentration of test compounds, also in concentrations that are higher than would be compatible with *in vivo* administration. Importantly, the concentrations of the test substances should ideally be applied in physiologically relevant concentrations to obtain as translational results as possible; however, this is challenging as luminal nutrient concentrations are highly variable and often the relevant vascular concentrations of certain test substances are unknown. Our approach is to study a spectrum of concentrations to look for any effect, and secondarily reconsider and adjust to expected physiological levels. For local effects associated with paracrine interactions in the gut, higher concentrations may be relevant. The problems with whole body metabolism of hormones, test compounds, and nutrients, and first-pass effects occurring in the liver that would be observed *in vivo* are avoided. The constant single-pass perfusion enables products and metabolites to be removed so that no accumulation (product inhibition) or recirculation occurs. This allows for accurate measurement and manipulation of intestinal absorption making it possible to distinguish whether the stimulus for secretion occurs before, during, or after intestinal absorption. The procedure requires a certain level of surgical skills and is unsuitable for screening purposes as it is laborious and relatively expensive considering the cost of perfusion buffer and animals. On the other hand, the time resolution is unsurpassed, and comparative studies of different compounds and/or doses can easily be made during several hours of perfusion. Intracellular signaling can only be studied indirectly with this model, and the effects might be exerted via a neighboring cell or enteric neurons. The perfusion studies therefore need to be complemented with other models such as primary intestinal cell cultures, cell lines, intestinal organoids, or Ussing chambers as reviewed in detail elsewhere [73–75]. The benefits and limitations of each of the mentioned models (primary intestinal cell cultures, cell lines, intestinal organoids, or Ussing chambers) were discussed in detail previously [75]. The main limitation of the perfusion system is that although it is possible to study also intracellular mechanisms and pathways, it is difficult to assign any changes after “intracellular” interventions to a specific cell type, since all cells in the preparation will be affected. On the other hand, if a mechanism identified in isolated cell systems does not result in changes in secretion in the perfusion model, its physiological relevance may be questioned. Another major limitation of the perfusion model is that mechanisms associated with absorption of long-chain fatty acids are studied with difficulty, as lymph draining system is disrupted in the model. It must be noted that the perfusion model is a rodent model and species differences may exist, as exemplified by sulfonylureas stimulating GLP-1 secretion in rodents [76] but not in humans [77], thus any potential clinical relevance should be confirmed in human studies. Nevertheless, the discrepant findings regarding the sulfonylureas may also reflect that the doses used for preclinical studies are often much higher and the difference may reflect different degrees of closure of the K_ATP_ channels rather than a species difference.

The remainder of this mini-review will focus on how the isolated perfused intestine has been used in our group as a model to investigate the nutrient-sensing mechanisms of EECs with a focus on the GLP-1 secreting cells. Nutrient-stimulated GLP-1 secretion has been reviewed elsewhere [1,73,75,78–80].

### Identifying the sensing and release mechanisms of EECs using the isolated perfused intestine as a model

#### Carbohydrates

The release of GI hormones occurs as nutrients reach and are absorbed across the intestinal epithelium. Carbohydrates are ingested as polysaccharides, oligosaccharides, disaccharides, and monosaccharides. All carbohydrates require digestion into monosaccharides (glucose, galactose, and fructose) by digestive enzymes and brush border enzymes before they are absorbed by the enterocytes and the EECs.

In the perfused rat intestine, luminal (but not vascular) glucose stimulates GLP-1 (PYY and GIP [81]) secretion in an absorption-dependent manner [71]. When investigating the mechanisms, it was found that inhibition of voltage-gated calcium channels reduced glucose-induced GLP-1 secretion. A reduction was also observed when a K_ATP_ channel opener was administered and when ATP synthesis was inhibited [71]. Further supporting a role for K_ATP_ channel involvement, closure of these channels by sulfonylurea administration stimulated GLP-1 secretion [71]. This is not observed clinically *in vivo* [82], most likely because the clinical doses of sulfonylureas produce a limited closure of the K_ATP_ channels [83]. The perfusion model allows extensive K_ATP_ channel closure and thus provides clarity about such a mechanism. When the perfusion buffer was depleted from sodium, the glucose-induced GLP-1 response was inhibited; however, blockage of voltage-gated sodium channels had no effect [71]. Glucose-induced GLP-1 secretion was completely lost in the presence of a sodium/glucose cotransporter 1 (SGLT1) inhibitor and stimulation with a nonmetabolizable SGLT1 substrate significantly increased GLP-1 secretion [71]. However, comparing the effects of a specific SGLT1 substrate with those elicited by glucose, glucose alone induced a greater GLP-1 response, pointing to the existence of additional SGLT-1 independent mechanisms for glucose-stimulated GLP-1 secretion [71]. A possible mechanism was proposed to be translocation of basolateral glucose transporter 2 (GLUT2) as GLUT2-specific inhibitor significantly reduced, but did not eliminate, glucose-stimulated GLP-1 secretion [71]. In the perfused rat intestine, artificial sweeteners were found not to be a potent GLP-1 stimulus [71,81]. Taken together, these data suggest that glucose is absorbed by GLP-1-producing EECs, via SGLT1. The accompanying sodium ions depolarize the cells, but ATP generation, with a resulting closure of K_ATP_ channels, may contribute to the depolarization, which leads to calcium influx, and finally, GLP-1 release (Figure 1).

#### Proteins

A protein-rich diet is in theory an advantageous diet for people living with obesity due to its satiety effect, which has been shown to exceed that of equicaloric carbohydrate and fat-rich meals [84], even though this is a debated [85]. Proteins are digested into oligopeptides, tri-, and di-peptides, and eventually more than 20 individual amino acids. This results in a wide repertoire of digestive products making it challenging to study protein sensing by EECs. One way is by using enzymatically generated protein digestion products derived from meat containing a nonspecific mix of oligopeptides and amino acids called peptones as a stimulus. In the perfused rat intestine, peptones stimulate secretion of GLP-1, PYY, neurotensin, CCK, and GIP [21].

Using the isolated perfused proximal small rat intestine, it was established that luminally infused amino acids were rapidly absorbed in the intestine (as reflected by a rise in vascular amino acids) and that amino acids applied from both the luminal and vascular side stimulated GLP-1 release [29]. When absorption of di- and tri-peptides were inhibited by blocking peptide transporter 1, the peptone-induced GLP-1 response was almost eliminated thus revealing the importance of this pathway and that absorption seems to be essential [29]. When inhibiting the voltage-gated calcium channels, no change in peptone-mediated GLP-1 release was observed, likewise a K_ATP_ channel opener did not affect amino acid-induced GLP-1 secretion, whereas a phospholipase C inhibitor decreased GLP-1 secretion [29]. Several amino acid receptors are present in GLP-1-secreting cells, including the calcium-sensing receptor (CaSR) [86,87]. Vascular, but not luminal, stimulation with a CaSR agonist stimulated GLP-1 secretion, suggesting that the receptor is located at the basolateral membrane [88]. Inhibiting CaSR, reduced the peptone-induced GLP-1 response, indicating a role for CaSR in sensing absorbed protein digestion products and stimulating GLP-1 secretion [88]. Amino acid activation of other amino acid receptors (GPR35, GPR93, GPR142, and the umami taste receptors (Tas1R1/Tas1R3)) did not result in GLP-1 secretion [88]. Further investigation of amino acid sensing mechanisms using the isolated perfused proximal small rat intestine revealed that the individual amino acids differ markedly in their capacity to stimulate GLP-1 release. In addition, the mechanism of stimulation differs between the individual amino acids as some amino acids stimulated GLP-1 secretion only when infused vascularly (l-arginine and l-tryptophan), whereas others only stimulated secretion when infused luminally (l-valine and l-glutamine) [88]. l-Valine was the most potent luminal stimulator of GLP-1 release [88]. Because of this, l-valine-induced GLP-1 secretion was further investigated in the isolated perfused proximal small rat intestine [89]. Blocking voltage-gated calcium channels blunted the l-valine-induced GLP-1 response and administration of a K_ATP_ channel opener also inhibited the l-valine-induced GLP-1 response [89]. When luminal saline solution was replaced with a sodium-deprived buffer, the GLP-1 response was not affected [89]. Based on these results, it was suggested that intracellular metabolism of l-valine, leading to the production of ATP and closure of K_ATP_ channels, membrane depolarization and activation of voltage-gated calcium channels is the mechanism of l-valine-induced GLP-1 secretion [89]. In this study, l-valine was found also to increase both GLP-1 and PYY secretion from the perfused rat colon [89]. Taken together, these data suggest that multiple release mechanisms exist for amino acid-induced GLP-1 secretion, and that the mechanisms resulting in EEC secretion vary depending on the amino acid.

#### Lipids

Fat is ingested mainly in the form of triglycerides, phospholipids, or cholesterol esters. These lipids are nonabsorbable but are digested by pancreatic lipases to free fatty acids, monoacylglycerols, lysophopholipids, and cholesterol. The digestive products join with bile acids to form micelles, enabling transportation across the hydrophilic mucus layer covering the apical membrane of the intestinal cells. Once in the intestinal cells, the long-chain fatty acids are re-esterified and incorporated into chylomicrons which are taken up by the lymphatic system after exocytosis from the enterocytes. In contrast, medium-chain fatty acids and short-chain fatty acids can passively diffuse across the apical and basolateral membrane. The lymphatic drainage makes it challenging to study long-chain fatty acid absorption using the isolated perfused intestine as a model, and other methods allowing lymph drainage may be needed to study the effects of these. Postabsorption effects of the lipids can be studied by vascular administration of the lipids; however, the variation in lipid species further challenges the study of the sensing mechanisms of lipids.

In the perfused proximal small rat intestine, vascular administration of the endogenous long-chain free fatty acid receptor-1 (FFAR1) ligand, linoleic acid, and four synthetic FFAR1 agonists increased GLP-1 secretion whereas luminal administration was ineffective [28]. Thus, the stimulation of the FFAR1 appears to require ligand absorption prior to stimulating GLP-1 secretion [28]. Short-chain fatty acids are generated in the colon by fermentation of nondigestible fibers and are thought to stimulate GLP-1 secretion by activation of the colonic short-chain fatty acid receptors; FFAR2 and FFAR3 [90]. However, in the perfused rat colon, short-chain fatty acids did stimulate colonic GLP-1 secretion with moderate efficacy and not through FFAR2 or FFAR3 activation as neither agonizing nor antagonizing these receptors influenced GLP-1 secretion, suggesting that short-chain fatty acids seem to increase GLP-1 secretion secondarily to their intracellular metabolism [91].

## Conclusion

This mini-review briefly describes our group’s experiences with the isolated perfused intestine as a model to investigate the nutrient-sensing mechanisms of EECs with a focus on the GLP-1-secreting cells. Importantly, several other studies using different models have been conducted to investigate sensing mechanisms and secretion of EECs and these perfusion studies do not stand alone. Their strength is their physiological relevance, but for the full picture combinations of perfusion and single cells studies are required and should be employed in future studies.

**Perspectives**

- The use of the isolated perfused intestine as a model to investigate the sensing mechanisms and secretion patterns of EECs is highly relevant, because increasing the secretion of the GI hormones is of translational interest in metabolic diseases such as obesity and type 2 diabetes.
- The concept of one cell–one hormone no longer holds and it is therefore of interest to study the integrated responses of the regional EECs to physiological and nutritional stimuli.
- To fully establish the sensing mechanisms and secretion patterns of EECs, perfusion studies and single-cell studies should be combined.

## Abbreviations

CCK: cholecystokinin
CaSR: calcium-sensing receptor
EEC: enteroendocrine cell
FFAR1: free fatty acid receptor-1
GI: gastrointestinal
GIP: glucose-dependent insulinotropic polypeptide
GLP-1: glucagon-like peptide 1
GLP-2: glucagon-like peptide 2
GLUT2: glucose transporter 2
PYY: peptide YY
RYGB: Roux-en-Y Gastric Bypass
SGLT1: sodium/glucose cotransporter 1
